# Solvent-Free Synthesis of Modified Pectin Compounds Promoted by Microwave Irradiation

**DOI:** 10.3390/molecules171012234

**Published:** 2012-10-18

**Authors:** Enrica Calce, Valeria Bugatti, Vittoria Vittoria, Stefania De Luca

**Affiliations:** 1 Institute of Biostructures and Bioimages, National Research Council, 80138 Naples, Italy; 2 Department of Industrial Engineering, University of Salerno, 84084 Fisciano (SA), Italy

**Keywords:** pectin, microwave irradiation, solvent-free, green chemistry

## Abstract

Microwave-assisted solvent-free modification of pectin was successfully accomplished, consisting in the esterification of several fatty acids by pectin alcoholic functions. The reaction was performed by simply mixing the reagents with a catalytic amount of the inorganic base (potassium carbonate) and irradiating the obtained mixture with microwaves for a short time (3–6 min). The replacement of the traditional heating with a microwave source allowed the development of a new synthetic protocol which provided increased yield of the final products, since it eliminates the small amount of degraded polysaccharide produced during traditional oil bath heating. The desired esters were fully characterized by FT-IR spectroscopy and thermogravimetric analysis.

## 1. Introduction

The development of green synthetic protocols in order to reduce or eliminate the use and generation of hazardous substances constitutes one of the major challenges for chemists. In particular, there has been an increasing demand for efficient organic solvent-free synthetic process, however the ideal approach would be carrying out reaction in total absence of solvent, thus gaining the great advantage of low cost, low environmental impact and low toxicity in handling [1].

On the other hand, the field of microwave-assisted chemistry has experienced growth in the last years, becoming increasingly popular in industry as well as in academia. In fact, while the traditional heating equipment, like oil baths and heating jackets, often generate a temperature gradient with local overheating of the sample which can undergo decomposition, microwave irradiation is characterized by uniform heating throughout the material which allows high efficiency and improved reproducibility of the chemical process. Moreover, microwave-assisted chemistry generally reduces the unwanted side reactions and increases the purity of the final products [2,3].

In this context, the combination of microwave activation and solvent-free conditions in organic synthesis provides clean chemical processes characterized by enhanced reaction rates, higher yields and enhanced ease of manipulation [4,5]. The practical feasibility of microwave-assisted solvent-free protocols has been already demonstrated in useful transformations involving protection/deprotection, condensation, oxidation, reduction, rearrangement reactions and in the synthesis of various heterocyclic systems on inorganic solid supports [6,7].

We have recently developed a solvent-free synthetic process in order to modify pectin via acylation of alcoholic functions of the polysaccharide by using several fatty acid anhydrides [8,9]. The whole synthetic protocol uses naturally occurring starting materials and does not produce dangerous waste, nor byproducts. Our goal has been to generate new materials, with potential novel applications, by using renewable resources and limiting the number of chemicals and reaction steps [10,11,12,13,14]. Indeed natural polymers suffer from low water resistance and poor mechanical properties, and these drawbacks are limiting factors for their use as manufactured materials. The chemical modification with fatty acids can improve water resistance and barrier properties, opening new possibilities in packaging applications. Moreover the extent of modification can widely modulate the pectin physical properties.

As next step we tried to switch from the traditional heating employed to promote the above mentioned reaction to a microwave assisted protocol. Herein, we describe our results on this environmentally friendly microwave approach for the synthesis of several pectin-derived compounds.

## 2. Results and Discussion

Pectins are important polysaccharides extracted from the cell walls of most plants. Apple pomace represents one of the major sources of commercial pectin and has applications in food, pharmaceutical and medical industries, in drug delivery, as a paper substitute, and in foams and plasticizers, *etc.* [15,16,17]. From a structural point of view, pectins are anionic polysaccharides consisting primarily of 1,4-α-D-galacturonosyl units and their methyl esters, interrupted in places by 1,2-α-L-rhamnopyranosyl units [18,19].

In a previous paper we described a process for the functionalization of apple pectin with several naturally occurring fatty acids [8,9]. They were activated as anhydrides and esterified by pectin alcoholic groups in the presence of catalytic amounts of K_2_CO_3_. The reaction took place under solvent-free conditions, just by milling all reagents and heating the obtained mixture at 160 °C.

**Scheme 1:**
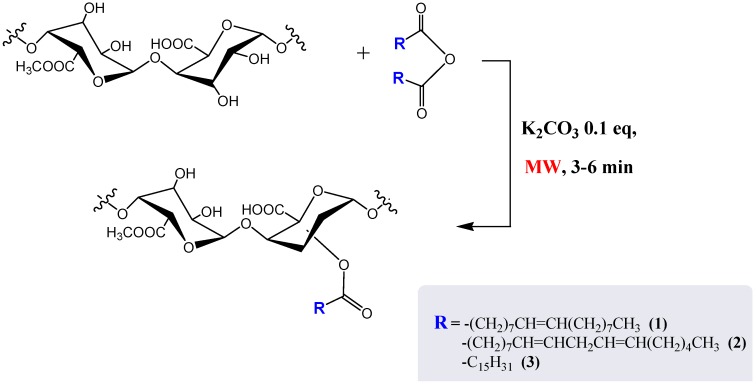
Synthetic strategy for pectin functionalization.

In this paper we report a study conducted on the above mentioned solvent-free reaction by replacing the traditional oil bath heating with microwave irradiation of the neat reactants. In particular, all esterification reactions were carried out by mechanical milling of the polysaccharide with the appropriate fatty acid anhydrides in the presence of a catalytic amount of base (K_2_CO_3_) and few drops of ethanol (Scheme 1).

Due to the polar characteristic of this solvent, the aim was to increase the dielectric properties of the reaction medium in order to allow sufficient heating by microwaves [7]. Next, the obtained mixture was irradiated with microwaves (900 W) for one (compounds **1a**, **2a**, **3a**) or two cycles (compounds **1b**, **2b**, **3b**) of 3 min each (Table 1).

**Table 1:** Pectin derivatives synthesized by microwave irradiation.

Compound	Fatty acid	Time(min)
**1a**	Linoleic acid	3
**1b**	Linoleic acid	6
**2a**	Oleic acid	3
**2b**	Oleic acid	6
**3a**	Palmitic acid	3
**3b**	Palmitic acid	6

After cooling, the crude product was washed with ethyl acetate in order to remove all unreacted fatty acids, then the solid was titrated with 0.5 N HCl until a neutral pH was reached and the resultant salt KCl was removed by dialysis in Milli-Q water. After lyophilization, the final products were fully characterized by FT-IR spectroscopy and, in a subsequent step, their thermogravimetric properties were analyzed.

### 2.1. Infrared Analysis

**Figure 1:**
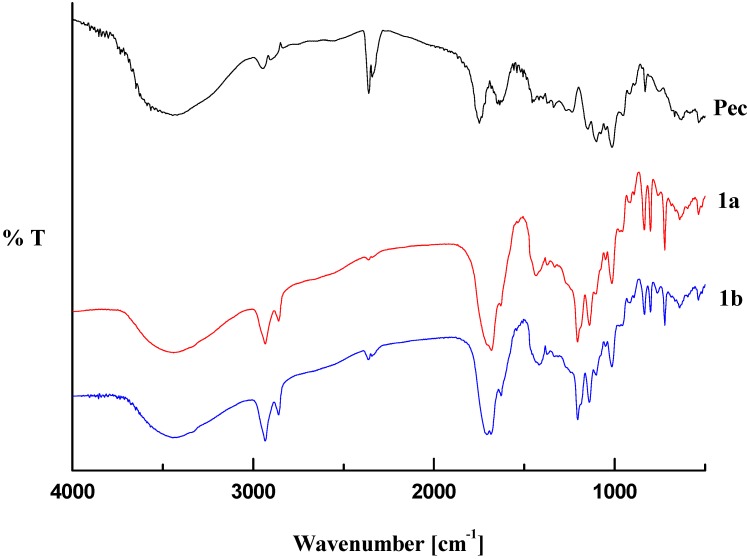
Transmittance FT-IR spectra pectin-linoleate (**1**) and pectin.

**Figure 2:**
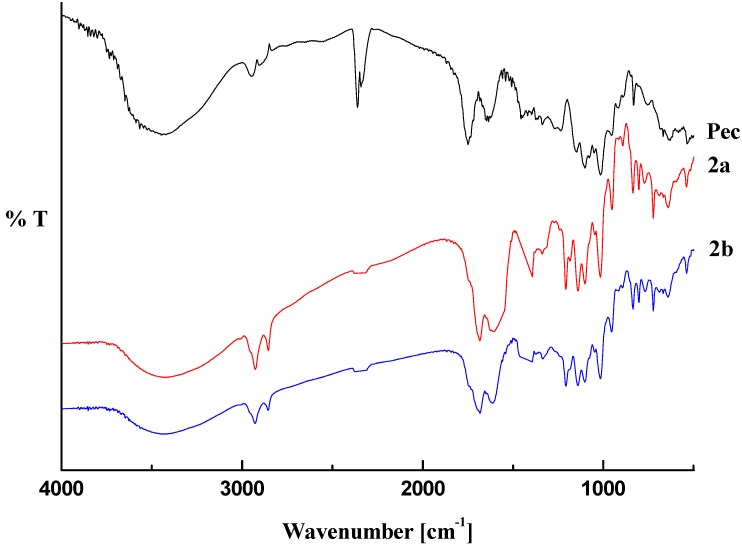
Transmittance FT-IR spectra pectin-oleate (**2**) and pectin.

**Figure 3:**
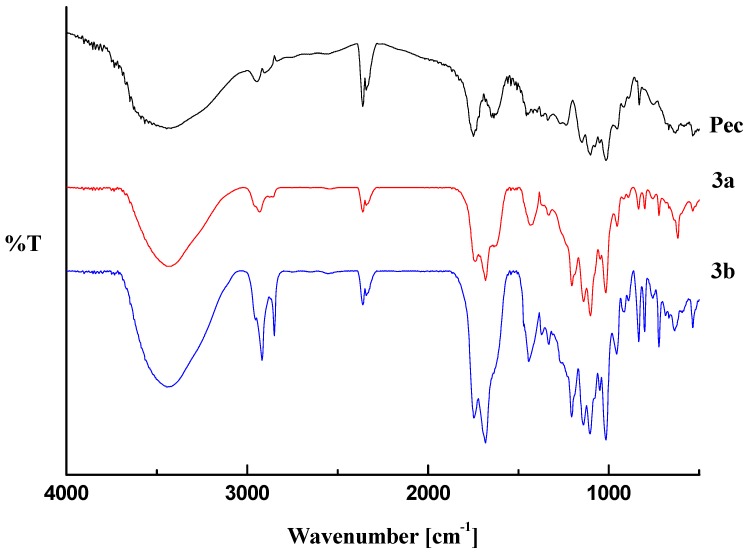
Transmittance FT-IR spectra pectin-palmitate (**3**) and pectin.

A general view of the FT-IR spectra of pectin-linoleate (compounds **1a**,**b**) pectin-oleate (compounds **2a**,**b**) and pectin-palmitate (compounds **3a**,**b**) are shown in Figure 1, Figure 2, Figure 3. For comparison purposes a spectrum of the native pectin is also reported in each figure [20].

The obtained spectral data were analyzed by comparing the following characteristic regions: O–H stretching (3100–3600 cm^−1^); C–H stretching (2800–3000 cm^−1^); carboxylic group stretching region (1200–1800 cm^−1^) [21,22,23] and the “fingerprint” region (under 2000 cm^−1^) which reflects the monosaccharide composition of the analyzed pectin [24,25].

The infrared analysis of the modified pectin samples revealed that the main changes occurred in those regions accounting for the chemical modifications introduced into the polysaccharide. In major details, a decrease of the O–H stretching band (in the 3500–3200 cm^−1^ range) is related to the decrease of the hydroxyl pectin groups upon reaction with the fatty acid anhydrides. On the other hand, an increase in the C–H stretching region (2931–2934 cm^−1^ for pectin-linoleate; 2926 cm^−1^ for pectin-oleate; 2919–2926 cm^−1^ for pectin-palmitate) and the appearance of well defined band (2854 cm^−1^ for pectin-linoleate; 2849–2855 for pectin-oleate; 2847–2850 cm^−1^ for pectin-palmitate) was also observed.

The region featuring the carboxylic groups (1750–1350 cm^−1^) is the more interesting. Concerning the FT-IR spectrum of the pectin, it shows three peaks at 1747, 1649, 1452 cm^−1^ in the carboxylic groups region assigned to the C=O stretching of methylated carboxyl groups, and to anti-symmetric and symmetric stretching modes of COO^−^, respectively [20]. For each sample the appearance of a new band in the C=O ester stretching region, which overlaps with the pectin methyl ester band, accounted for the esterification of the fatty acid employed (1700–1705 cm^−1^ for pectin-linoleate; 1695–1700 cm^−1^ for pectin-oleate; 1698–1705 cm^−1^ for pectin-palmitate) [13]. Also the band in the COO^−^ antisymmetric stretching region (1620–1630 cm^−1^) was partially overlapped. Moreover, for each synthesized pectin derivative an increased intensity of the band at 1410–1433 cm^−1^ was observed. In this regard, it is worth remembering that this region does not account, in a quantitative manner, for the pectin free carboxylic groups [26].

Other changes are registered in the C–O stretching region at 1300–1100 cm^−1^; in particular new bands at 1207 cm^−1^ and 1142–1133 cm^−1^ were observed in the spectrum of each sample. Concerning the “fingerprint region” (1100–1000 cm^−1^), no significant changes were observed for any analyzed sample. The 850–600 cm^−1^ region contains new bands which are related to the introduced fatty acid chains [27,28].

### 2.2. Thermal Analysis

All of the samples present a characteristic three-step thermal degradation, typical of pectin [29,30]. In pure pectin, the first step, occurring at about 80 °C, corresponds to the water loss and can be evaluated as 12% of the initial mass; then, it is followed by the second step, between 200 and 400 °C. In this temperature range, it has been reported that the degradation, covering about 60% of mass loss, is primarily derived from pyrolytic decomposition. It consists in a primary and secondary decarboxylation involving the acid side group and a carbon in the ring. The third step between 500 and 700 °C corresponds to the oxidation region. 

**Figure 4:**
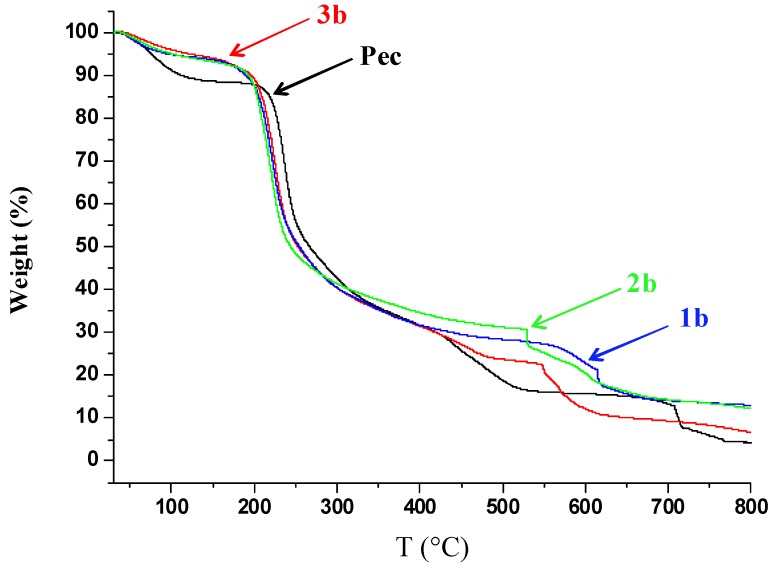
The loss of weight of the pristine and modified pectin samples **1b**, **2b**, and **3b** as a function of temperature.

In the modified samples of pectin with linoleic (**1b**), oleic (**2b**), and palmitic (**3b**) acids (Figure 4), it can be observed that the first weight loss due to the adsorbed water is consistently lower compared with that shown by the pristine pectin, giving a first indication that the presence of the fatty acid chain drastically reduces the adsorbed water under the same storage conditions as the pure pectin.

Moreover, the chemical modification produces an increase in the evaporation temperature, going from 80 °C for pristine pectin to higher values for all the three modified pectins. The water evaporation step is greatly reduced in these samples, indicating that a very small quantity of water is absorbed. The second degradation stage is very similar for either the pure or the modified pectin samples, although a slightly lower mass loss and a lower midpoint temperature are observed for the modified samples. The third stage, due to oxidative reactions is postponed, particularly for linoleic residues, as expected for the oxygen scavenging effect of the double bonds.

## 3. Experimental

### 3.1. Materials

Apple peel pectin was purchased from Fluka (Buchs, Switzerland). It is a powder sample with high molecular weight (30,000–100,000 g/mol) and a high degree of esterification (70–75%) on a dry basis. The fatty acids and all solvents were purchased from Sigma-Aldrich (Buchs, Switzerland). 

### 3.2. Methods

Reactions were carried out in a domestic 900 W (2450 MHz) microwave oven (DeLonghi, Treviso, Italy). The FT-IR spectra were recorded on a Perkin-Elmer spectrometer, model Vertex 70. Samples were ground into a fine powder using an agate mortar before being compressed into KBr discs. The characteristic IR transmission spectra peaks were recorded at a resolution of 4 cm^−1^ over the 600–4000 cm^−1^ wavenumber region. Thermal analysis (TGA) was carried out in air atmosphere with a Mettler TC-10 Thermobalance (Novate Milanese, Italy) from room temperature to 1000 °C at a heating rate of 10 °C/min on 10 mg samples in duplicate. 

### 3.3. Synthesis of Modified Pectin Samples

Using an agate mortar, pectin (30 mg) was manually milled with the appropriate fatty acid anhydride (30 mg) in the presence of K_2_CO_3_ (0.1 equiv.) to obtain the different pectin-derived materials. Samples were irradiated with microwaves (900 W) for one or two cycles of 3 min each. After cooling to room temperature, the final crude product was washed with ethyl acetate. The obtained solid was dissolved in water, and 0.5 N HCl was titrated into the final solution until a neutral pH was reached. This solution was then dialyzed (membrane cut off 6000–8000) for one day in Milli-Q water and finally lyophilized to give the desired product. All modified pectin samples were analyzed by FT-IR spectroscopy. The bands relevant for the structural organization are: pectin-linoleate (**1**): FT-IR (cm^−1^): 3434 *ν* (O–H), 2930 and 2854 *ν* (C–H), 1732 *ν* (C=O methyl ester), 1700 *ν* (C=O fatty acid ester), 1629 *ν*_as_ (COO^−^), 1436 *ν*_as_ (COO^−^), 1207 and 1133 *ν* (C–O); pectin-oleate (2): FT-IR (cm^−1^): 3437 *ν* (O–H), 2926 and 2855 *ν* (C–H), 1750 *ν* (C=O methyl ester), 1695 *ν* (C=O fatty acid ester), 1621 *ν*_as_ (COO^−^), 1410 *ν*_as_ (COO^−^), 1207 and 1142 *ν* (C–O); and pectin-palmitate (3): FT-IR (cm^−1^): 3440 *ν* (O–H), 2919 and 2847 *ν* (C–H), 1750 *ν* (C=O methyl ester), 1698 *ν* (C=O fatty acid ester), 1629 *ν*_as_ (COO^−^), 1433 *ν*_as _(COO^−^), 1207 and 1142 *ν* (C–O).

## 4. Conclusions

A synthetic process for obtaining pectin-derived compounds has been described, consisting in functionalizing the alcoholic groups of the polysaccharide with several natural fatty acids. The reported procedure uses microwaves for activation and solvent-free conditions, and offers the advantages of being more efficient, cleaner and faster with respect the previously reported methodology, which uses traditional oil bath heating. Moreover, the microwave irradiation, allowing a uniform increase of the temperature throughout the sample, leads to less by-products and reagent decomposition, thus increasing the yield of the final products. The thermogravimetric analysis revealed for each modified pectin sample a degradation path very similar to that of the pristine pectin. In future, we plan to develop a microwave-assisted protocol which shall allow us to obtain, for each synthesized pectin derivative, compounds characterized by different degrees of substitution. 

## Supplementary Materials

Supplementary materials can be accessed at: http://www.mdpi.com/1420-3049/17/10/12234/s1.
