# Supplementary figures and images for: Solvent-Free Synthesis of Modified Pectin Compounds Promoted by Microwave Irradiation
Source: Molecules. 2012 Oct 18;17(10):12234–42. doi: 10.3390/molecules171012234 (PMC6268555; doi:10.3390/molecules171012234)

pectin-linolate  
Pulse Sequence: s2pu1

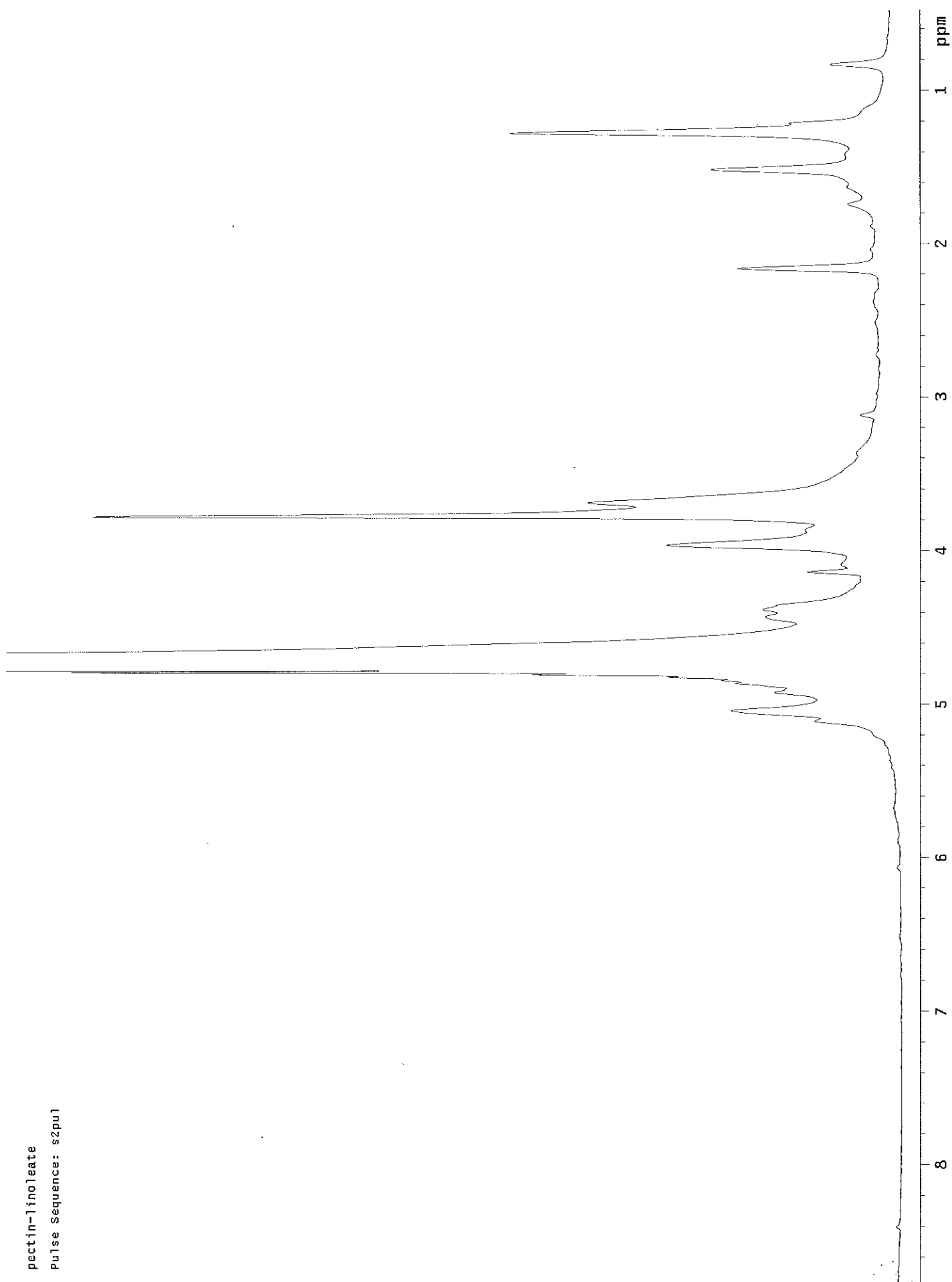

Supplement: Supplementary file 1 [file molecules-17-12234-s001.pdf]
